# Prolonged perceived stress and saliva cortisol in a large cohort of Danish public service employees: cross-sectional and longitudinal associations

**DOI:** 10.1007/s00420-017-1241-z

**Published:** 2017-07-11

**Authors:** Sigurd Mikkelsen, Julie Lyng Forman, Samuel Fink, Marianne Agergaard Vammen, Jane Frølund Thomsen, Matias Brødsgaard Grynderup, Åse Marie Hansen, Linda Kaerlev, Henrik Albert Kolstad, Reiner Rugulies, Jens Peter Bonde

**Affiliations:** 1 Department of Occupational and Environmental Medicine, Bispebjerg University Hospital, Copenhagen, Bispebjerg Bakke 23, 2400 Copenhagen, Denmark; 2 Section of Biostatistics, Department of Public Health, University of Copenhagen, Copenhagen, Denmark; 3 Department of Public Health, University of Copenhagen, Copenhagen, Denmark; 4 National Research Centre for the Working Environment, Copenhagen, Denmark; 5 Research Unit of Clinical Epidemiology, Institute of Clinical Research, University of Southern Denmark, Odense, Denmark; 6 Center for Clinical Epidemiology, Odense University Hospital, Odense, Denmark; 7 Department of Occupational Medicine, Danish Ramazzini Centre, Aarhus University Hospital, Århus, Denmark; 8 Department of Psychology, University of Copenhagen, Copenhagen, Denmark

**Keywords:** Cohen’s perceived stress scale, Diurnal cortisol, Chronic stress, Epidemiology, Occupational health, PRISME study

## Abstract

**Purpose:**

It is well known that acute stress can lead to a transient increase in cortisol secretion, but the effects of prolonged stress on cortisol secretion are uncertain. This study examines the cross-sectional and longitudinal associations between prolonged perceived stress and salivary cortisol.

**Methods:**

In 2007, 4467 Danish public service employees participated in a study of stress and mental health, and 3217 participated in a follow-up in 2009. Perceived stress during the past 4 weeks was assessed by Cohen’s four item perceived stress scale. Participants were asked to collect saliva 30 min after awakening and at approximately 20:00 in the evening. The cortisol dependence on perceived stress was examined in regression analyses adjusted for effects of potential confounders. We adjusted for a large variation in saliva sampling times by modelling the time trajectory of cortisol concentrations in the morning and in the evening and examined if they were influenced by perceived stress.

**Results:**

Perceived stress had no statistically significant effects on the level or time trajectory of morning or evening cortisol, neither cross-sectionally nor longitudinally. The 1 month prevalence of frequently perceived stress was low, approximately 2.5%.

**Conclusion:**

Our results did not support the hypothesis that prolonged perceived stress is associated with the level or time trajectory of morning or evening salivary cortisol.

**Electronic supplementary material:**

The online version of this article (doi:10.1007/s00420-017-1241-z) contains supplementary material, which is available to authorized users.

## Introduction

Stress may be defined as the response to exposures (stressors) that threaten the internal stability of the organism (Rosmond 2005). Psychological stressors may be defined as psychosocial exposures that are perceived as threatening or excessively demanding (Allen et al. 2014). It is well known that the acute stress response to psychosocial stressors involves activation of the hypothalamic–pituitary–adrenal (HPA) system with a large increase in adrenal secretion of cortisol within minutes (Allen et al. 2014; Dickerson and Kemeny 2004; Foley and Kirschbaum 2010), but further research is needed to examine if and how exposure to frequent or chronic psychological stressors affects HPA activity.

Alterations in HPA function may be reflected in changes in the normal level and diurnal trajectory of cortisol secretion. Salivary cortisol increases rapidly upon awakening to reach a peak level after 30–40 min, followed by a gradual decline to the lowest levels in the evening and the first hours of sleep, and then increases gradually during the hours before awakening (Karlamangla et al. 2013; Ranjit et al. 2005; Wilhelm et al. 2007). Changes in the level and diurnal pattern of cortisol secretion have been associated with a number of different somatic and mental disorders (Chrousos and Kino 2007; Kudielka and Wust 2010; McEwen 1998; Tsigos and Chrousos 2002), for example hypertension (Wirtz et al. 2007), ischaemic heart disease (Bhattacharyya et al. 2008; Kumari et al. 2011; Matthews et al. 2006), cancer (Abercrombie et al. 2004; Sephton et al. 2000), depression (Kudielka and Wust 2010; Stetler and Miller 2011), and upper respiratory illness (Edwards et al. 2003; Smyth et al. 1997), all of which have also been associated with exposure to psychological stressors (Bonde 2008; Cohen et al. 1991; Cuffee et al. 2014; Inoue 2014; McGregor and Antoni 2009). Thus, adverse health effects of frequent acute or chronic perceived stress could possibly be mediated through the HPA-axis. In particular, it is of interest to unravel if prolonged psychological stress is associated with persistently elevated levels of cortisol because of the well-known deleterious effects of even mild sustained hypercortisolism (Goddard et al. 2015).

Cohen’s perceived stress scale (PSS) is one of the most commonly used research tools for measuring perceived stress (Lee 2012). The PSS measures the degree to which situations in one’s life are appraised as unpredictable, uncontrollable, and overloading (Cohen et al. 1983). It was developed to meet the view that threats or challenges from life events, e.g., unemployment or bereavement, are appraised differently by different persons depending on their experience and coping resources, and was found to be a better predictor of measures of health and health-related outcomes than scales that assessed the number of stressful life events (Cohen et al. 1983).

A recent review on the relation between PSS and cortisol secretion found inconsistent results. However, most of the studies were fairly small and used different measures of cortisol (Halford et al. 2011). To our knowledge, no studies have yet examined the longitudinal association between PSS and cortisol secretion.

This study examined the association between perceived stress measured by the four item version of the PSS (PSS-4) and the level and time trajectory of morning and evening salivary cortisol in a large population of public sector employees, repeated after 2 years. We hypothesized that cortisol in saliva would increase with increasing perceived stress, and examined this hypothesis cross-sectionally and longitudinally.

## Methods

### Study population and setting

In January 2007, we invited 10,036 public sector employees in Aarhus, Denmark to participate in a study on stress and mental health, the PRISME study (Kolstad et al. 2011). They were asked to fill in a questionnaire on psychosocial working conditions, health and other personal factors. They were further asked to collect saliva in two sampling tubes, one in the morning and one in the evening, and to record the sampling circumstances in a sampling log. A total of 4467 (45%) returned saliva sample material in 2007. In January 2009, participants from 2007 were asked to participate in a repetition of the baseline study. A total of 3217 (72%) returned saliva sample material.

### Perceived stress

We measured perceived stress by a Danish version of the PSS-4. The items, response categories, and scale score calculation are shown in Table 1.

**Table 1:** Four-item version of the perceived stress scale (PSS-4)

During the last 4 weeks how often…
1. Have you felt that you were unable to control the important things in your life?
2. Have you felt confident about your ability to handle your personal problems?
3. Have you felt that things were going your way?
4. Have you felt difficulties were piling up so high that you could not overcome them?

Response scores: 0 = never, 1 = almost never, 2 = sometimes, 3 = fairly often, and 4 = very often. Items 2 and 3 were scored in the reverse direction. Scale scores were calculated as the mean of item scores

### Cortisol measurements

Written instructions on how to sample, label, keep, and return saliva samples were included in the invitation letters together with two Salivette^®^ cotton tubes. Participants were asked to collect saliva 30 min after awakening and at approximately 20:00, preferably on a workday, and to note the time of sampling on the enclosed label.

The instruction emphasized that swabs should be kept in the mouth until thoroughly saturated and then in a refrigerator until they were returned by mail. The instructions did not ask for behavioral restrictions before saliva sampling (e.g., refraining from smoking, tooth brushing, eating, drinking, and physical activity). The samples were stored at −20 °C and analyzed within 10 months. Cortisol concentrations in saliva were analyzed with the Spectria Cortisol Coated Tube RIA (Orion Diagnostica, Finland). To show equivalence between different runs, natural saliva samples (5.5 and 22.7 nmol/l in 2007/5.9 and 24.2 nmol/l in 2009) were used as control materials. Between-run coefficients of variation (CVs) were 19% at 11.5 nmol/l and 16% at 49.2 nmol/l (Hansen et al. 2003). Further methodological details have been published previously (Hansen et al. 2003; Vammen et al. 2014).

### Potential confounders

A large number of factors may influence PSS (Lee 2012) and cortisol secretion (Kudielka et al. 2009; Stalder et al. 2016). We included the following factors as potential confounders (analytic categories in parenthesis): age, gender, socioeconomic factors [vocational education after school (<3 years, 3–4 years, >4 years), personal income], life style [smoking (present smoker (yes/no)), weekly alcohol consumption (>14 units of approximately 12.5 g of alcohol (yes/no)), leisure time physical activity (low/high), body mass index (BMI = weight (kg)/height^2^ (m))], general health (excellent or very good/good/fair or poor), ever diagnosed with a depression or anxiety disorder (yes/no), ever diagnosed with a cardiovascular disease (yes/no), disturbed sleep during the last 4 weeks [Karolinska Sleep Questionnaire (Hansen et al. 2012)], sleep duration of the night before saliva sampling, usual work schedule (daytime vs other schedules), working on the day of saliva sampling (yes/no), awakening time, and saliva sampling times (time since awakening for morning samples, clock time for evening samples).

### Data editing

We made a detailed review of the consistency of dates and times on the saliva sample tube labels compared to sample log recordings of dates and times and excluded samples with inconsistent or missing data. For a morning value to be valid, we further required that the sample was taken within 2 h from awakening and before 12:00, and for evening values that the sample was taken after 17:00. Cortisol concentrations above 100 nmol/l were considered to be outliers and were excluded. We excluded pregnant participants and participants with a missing PSS score. The final material for crude analyses of the cross-sectional relation between PSS and morning cortisol consisted of 3616 (81%) and 2494 (78%) persons in 2007 and 2009, respectively. The corresponding figures for evening cortisol were 4002 (90%) and 2819 (88%) persons. The material for longitudinal analyses included persons who participated in both examination rounds and consisted of 2121 persons for the morning cortisol analyses and 2597 persons for the evening cortisol analyses.

### Analysis

We analyzed the morning and evening cortisol as dependent variables with perceived stress as the explaining variable.

We estimated the cross-sectional and longitudinal effects of PSS on cortisol using the method described in Fitzmaurice et al. (2011). The cross-sectional effect is the effect of the average PSS on average cortisol across the two examination rounds, and the longitudinal effect is the effect of the change in PSS on change in cortisol from 2007 to 2009. These effects were estimated as independent effects in a linear mixed model further controlling for examination round, potential confounders, and for repeated measurements. The model utilizes all cross-sectional and longitudinal data in the same analysis, thus allowing to test if differences exist between cross-sectional and longitudinal effects. This test is sometimes referred to as the *Hausman test for unmeasured confounders* as differences in the cross-sectional effect and longitudinal effects may indicate a bias in the cross-sectional effect due to uncontrolled confounding (Fitzmaurice et al. 2011).

The effects of PSS on cortisol were analyzed as linear effects and as categories of PSS scores to account for non-linear effects, specifically effects at higher levels of PSS. For these analyses, PSS was categorized as low, medium, and high levels of perceived stress by scores 0 to <1.5, 1.5 to <2.5, and 2.5 to 4.0, corresponding to item response categories ‘never’ or ‘almost never’, ‘sometimes’, and ‘fairly often’ or ‘very often’.

Preliminary to the analyses, cortisol concentrations were transformed to their natural logarithm to reduce skewness and to stabilize variation. The results are presented as back-transformed effect estimates and then become the ratio change in cortisol by a 1 unit of change of PSS or for a PSS category compared to the reference category.

We adjusted for potential confounders as described in the “Methods” section. Age, personal annual income, BMI, and sleep-variables were included as linear variables, and awakening time and the morning and evening sampling times as two-piece linear splines fitted to the data. The most likely inflexion points for the splines were estimated by non-linear regression.

To see if the increase and decrease in morning and evening cortisol were modified by PSS, we examined the effects of the multiplicative interaction terms between PSS and the slopes of the linear splines, both in terms of the cross-sectional and longitudinal effects. We further estimated the cortisol awakening response (CAR) and the morning increase and total area under the curve (AUCi and AUCt) (Stalder et al. 2016) from the estimates of the level and slopes in the main analyses and examined if they differed with the level of perceived stress.

To address potential reporting bias, we examined if saliva sampling times differed by levels of perceived stress and we conducted a simulation study to assess the impact of the reporting bias on the estimates of the relation between cortisol and perceived stress. In addition, we made assessments of the attenuation in the estimated relation between PSS and cortisol due to inaccuracy of the PSS-4 scale (Supplementary material, Appendix 1).

#### Sensitivity analyses

To avoid potential overadjustment, we made sensitivity analyses with a reduced set of potential confounders, excluding life style, health, sleep factors, and awakening, and saliva sampling times which might be antecedent or intermediate factors of the relation between PSS and cortisol.

We further examined if the results depended on the estimated spline inflexion points by changing them to their lower and upper 95% confidence limits.

Data were analyzed with SAS version 9.3 and 9.4 (SAS Institute Inc, Cary, NC, USA). Mixed models were analyzed with the HPMIXED procedure.

## Results

Population characteristics at baseline and at follow-up are shown in Table 2. Compared to participants in 2007, participants in 2009 reported a higher education, higher annual income, less smoking, higher physical activity, more daytime work schedule, and sampled saliva less frequently on a workday than on a day off. These changes were mainly due to a secular trend and less to selective loss to follow up (data not shown).

**Table 2:** Population characteristics at baseline and at follow-up

	2007 (*n* = 3616)		2009 (*n* = 2494)	
	*n*	%	*n*	%
Age				
19–29	297	8.2	86	3.5
30–39	855	23.6	544	21.8
40–49	1103	30.5	703	28.2
50–59	1198	33.1	921	36.6
60–69	163	4.5	240	9.6
Gender				
Female	2817	77.9	1953	78.3
Vocational education after school				
<3 years	704	19.5	401	16.3
3–4 years	2488	69.0	1729	69.6
>4 years	416	11.5	356	14.3
Annual income >300,000 DKK				
Yes	1668	48.6	1521	64.6
General health				
Excellent/very good	1826	50.8	1351	54.4
Good	1342	37.4	912	36.7
Fair/poor	421	11.7	220	8.9
Psychiatric disease, ever diagnosed				
Yes	549	15.2	389	15.6
Cardiovascular disease, ever diagnosed				
Yes	532	14.7	415	16.6
Current smoker				
Yes	647	18.0	326	13.2
Alcohol >14 units/week				
Yes	306	8.5	204	8.3
High physical activity in leisure time				
Yes	1675	46.5	1257	50.8
Body mass index				
<18.5	68	1.9	35	1.4
18.5–24.9	2294	64.0	1546	62.6
25.0–29.9	958	26.7	690	27.9
30+	263	7.3	200	8.1
Disturbed sleep, last 4 weeks (scores 1–5)				
Score ≥3	959	26.6	558	22.4
Daytime work schedule				
Yes	2329	65.8	1658	71.9
Saliva sampled on a workday				
Yes	3148	89.2	2040	83.94
Sleep duration last night (h)				
<6.5	7202	20.0	445	17.9
Awakening time				
02:55–06:00	1174	32.5	693	27.8
06:00–07:00	1656	45.8	1172	47.0
07:00–11:15	786	21.7	629	25.2

Table 3 shows the distribution of saliva sampling times. Sampling times were concentrated around 30 min after awakening and 20:00 in the evening in accordance with the instruction. However, there was a large variation across large time spans, and owing to the large study sample, there was a fairly large number of participants in even narrow categories of sampling times. This distribution allowed the estimation of the morning and evening cortisol trajectories as outlined above.

**Table 3:** Frequency distribution of morning and evening saliva sampling times in 2007 and 2009

Morning saliva sampling time (minutes after awakening)					Evening saliva sampling time				
Minutes	2007		2009		Clock time	2007		2009	
	*n*	%	*n*	%		*n*	%	*n*	%
0–4	67	1.9	55	2.2	17:00–18:59	48	1.2	44	1.6
5–9	50	1.4	50	2.0	19:00–19:29	85	2.1	54	1.9
10–14	62	1.7	51	2.0	19.30–19:44	151	3.8	86	3.1
15–19	89	2.5	80	3.2	19:45–19:59	235	5.9	163	5.8
20–24	130	3.6	89	3.6	20:00–20:14	1601	40.0	953	33.8
25–29	209	5.8	144	5.8	20:15–20:29	338	8.5	218	7.7
30–34	1274	35.2	825	33.1	20:30–29:44	304	7.6	190	6.7
35–39	460	12.7	302	12.1	20:45–20:59	132	3.3	77	2.7
40–44	323	8.9	221	8.9	21:00–21:14	257	6.4	205	7.3
45–49	285	7.9	214	8.6	21:15–21:29	58	1.5	45	1.6
50–55	118	3.3	65	2.6	21:30–21:44	117	2.9	100	3.6
55–59	65	1.8	44	1.8	21:45–21:59	48	1.2	31	1.1
60–64	164	4.5	117	4.7	22:00–22:14	174	4.4	179	6.4
65–74	62	1.7	37	1.5	22:15–22:44	153	3.8	138	4.9
75–84	91	2.5	64	2.6	22:45–23:14	130	3.3	139	4.9
85–94	78	2.2	68	2.7	23:15–23:44	76	1.9	89	3.2
95–120	89	2.5	68	2.7	23.45–02:30	95	2.4	108	3.8
Total	3616	100	2494	100	Total	4002	100	2819	100

The cross-sectional distribution of cortisol by categories of PSS is shown in Table 4. The highest PSS scores seem associated with a lower morning cortisol level in both examination rounds, but this trend was not statistically significant. Spearman correlation coefficients between PSS and morning cortisol were low and non-significant, −0.017 (*p* = 0.30) in 2007 and −0.015 (*p* = 0.46) in 2009. The corresponding correlation coefficients for evening cortisol were −0.004 (*p* = 0.78) and 0.030 (*p* = 0.11). Morning cortisol was significantly higher in 2009 than in 2007. The Spearman correlation coefficients of cortisol in 2007 and 2009 were 0.30 (morning) and 0.19 (evening). PSS scores were significantly higher in 2007 than in 2009. The Spearman correlation coefficient of PSS in 2007 and 2009 was 0.46. Cronbach’s alpha of PSS was 0.60 in 2007 and also in 2009. The distribution of PSS was skewed with only 2.0–2.5% of participants reporting PSS ≥ 2.50 corresponding to average item responses ‘fairly often’ or ‘very often’ during the previous month (Table 4).

**Table 4:** Morning and evening concentrations of cortisol in saliva (nmol/l), median, and 5–95% percentiles, by examination round and categories of perceived stress scores (PSS)

PSS	2007				2009			
			Cortisol (nmol/l)				Cortisol (nmol/l)	
	*n*	%	Median	5–95%	*n*	%	Median	5–95%
Morning								
0 to <0.50	407	11.3	11.4	3.6–25.2	389	15.6	13.2	4.3–27.8
0.50 to <1.50	2092	57.9	11.1	3.0–25.0	1518	60.9	13.9	4.2–30.0
1.50 to <2.50	1032	28.5	11.5	3.5–25.5	536	21.5	13.5	3.6–31.2
2.50 to 4.00	85	2.4	9.7	3.2–24.1	51	2.0	11.5	4.0–24.1
Total	3616	100	11.2	3.1–25.2	2494	100	13.6	4.1–29.2
Evening								
0 to <0.50	457	11.4	1.4	0.5–5.1	445	15.6	1.4	0.4–5.8
0.50 to <1.50	2297	57.4	1.4	0.4–5.4	1692	60.0	1.4	0.5–5.7
1.50 to <2.50	1143	28.9	1.4	0.4–6.1	623	22.1	1.5	0.4–6.4
2.50 to 4.00	105	2.6	1.5	0.4–5.4	59	2.1	1.3	0.4–4.8
Total	4002	100	1.4	0.4–5.6	2819	100	1.4	0.4–5.8

The morning and evening cortisol trajectory determined by the two-piece linear models for effects of sampling times showed an increase after awakening from 6.8 to 11.3 nmol/l (66% increase) in 2007 and from 8.6 to 13.7 nmol/l (59% increase) in 2009 with peak concentration 34 min after awakening in both examination rounds, and then a slower decrease. The evening concentrations decreased slowly after 17:00 to a trough at 20:45 in 2007 and at 21:15 in 2009, approximately 15 h after awakening, and then increased slowly. The diurnal variation in saliva cortisol is graphically illustrated in Supplementary material (Appendix 2).

The differences in cortisol from 2007 to 2009 by categories of PSS in 2007 and 2009 are shown in Table 5. In these crude data, there is no pattern indicating that a change to higher or lower PSS scores from 2007 to 2009 is related to lower or higher cortisol levels in 2007 compared to 2009. It is also noteworthy that approximately 56% remained in the same PSS category, 40% changed one category up or down, and 4% changed two or three categories. Only two participants changed three categories, and less than 0.5% of the participants reported PSS ≥ 2.50 corresponding to ‘fairly often’ or ‘very often’ in both examination rounds.

**Table 5:** Differences in cortisol concentrations from 2007 to 2009 by categories of PSS scores in 2007 and 2009

PSS 2007	PSS 2009	Morning			Evening		
			ΔCortisol^a^ (nmol/l)			ΔCortisol^a^ (nmol/l)	
		*n*	median	5–95% (nmol/l)	*n*	median	5–95% (nmol/l)
0 to <0.50		255			314		
	0 to <0.50	125	0.9	−10.5–16.9	141	−0.10	−2.7–3.3
	0.50 to <1.50	114	1.2	−13.0–15.9	151	0.00	−2.9–5.2
	1.50 to <2.50	16	4.3	−6.3–10.3	22	0.30	−3.6–4.4
	2.50 to 4.00	0	–	–	0	–	
0.50 to <1.50		1262			1520		
	0 to <0.50	179	1.5	−12.2–14.6	229	0.00	−3.0–3.6
	0.50 to <1.50	867	1.6	−12.8–17.0	1030	0.10	−3.3–3.8
	1.50 to <2.50	204	1.3	−13.9–18.4	247	0.20	−2.1–4.4
	2.50 to 4.00	12	3.2	−17.7–9.6	14	0.25	−2.4–4.0
1.50 to <2.50		559			701		
	0 to <0.50	31	1.9	−7.0–15.4	36	0.15	−1.5–4.3
	0.50 to <1.50	292	1.3	−12.4–16.1	354	0.10	−4.1–3.0
	1.50 to <2.50	215	1.4	−12.4–14.5	282	0.10	−3.9–3.9
	2.50 to 4.00	21	−2.2	−14.3–8.5	29	0.10	−3.6–3.3
2.50 to 4.00		45			62		
	0 to <0.50	2	6.4	6.2–6.6	2	−0.55	−0.8–−0.3
	0.50 to <1.50	15	4.7	−18.8–33.5	15	0.00	−1.6–10.4
	1.50 to <2.50	22	0.9	−5.4–11.7	33	0.00	−5.7–1.9
	2.50 to 4.00	6	1.5	−7.4–9.6	12	−0.60	−4.6–2.2
Total		2121			2597		

^a^Concentration in 2009 minus concentration in 2007

Table 6 shows the cross-sectional and longitudinal effects of PSS (see section on “Analysis”). There were no significant effects of PSS on levels of morning or evening cortisol. The Hausman test of no difference between cross-sectional and longitudinal effects was not significant in any of the models, indicating that it is not very likely that unmeasured confounding has affected the results.

**Table 6:** Cross-sectional and longitudinal effects of perceived stress (PSS) on morning and evening saliva cortisol concentrations

PSS	Crude model^a^				Adjusted model^b^			
	*N*	Effect ratio^c^	95% Cl	*p*	*N*	Effect ratio^c^	95% Cl	*p*
Morning								
Continuous								
Cross-sectional effect	6110	0.97	0.94–1.00	0.09	5226	1.03	0.99–1.07	0.14
Longitudinal effect	4242	1.01	0.94–1.05	0.87	3650	0.97	0.92–1.04	0.42
*p* ^d^				0.45				0.14
Categorical								
Cross-sectional effect								
0 to <1.50	4406	1			3797	1		
1.50 to <2.50	1568	0.99	0.94–1.04	0.64	1322	1.03	0.98–1.09	0.23
2.50 to 4.00	136	0.89	0.78–1.03	0.12	107	1.08	0.92–1.27	0.35
Longitudinal effect								
0 to <1.50	3142	1			2731	1		
1.50 to <2.50	1016	1.03	0.96–1.10	0.45	851	1.00	0.93–1.08	0.99
2.50 to 4.00	84	0.99	0.82–1.20	0.94	68	0.96	0.77–1.19	0.68
*p* ^d^				0.51				0.59
Evening								
Continuous								
Cross-sectional effect	6821	0.99	0.95–1.03	0.62	5727	0.98	0.94–1.03	0.49
Longitudinal effect	5194	1.06	0.99–1.13	0.08	4360	1.06	0.99–1.14	0.10
*p* ^d^				0.08				0.08
Categorical								
Cross-sectional effect								
0 to <1.50	4891	1			4141	1		
1.50 to <2.50	1766	1.01	0.96–1.07	0.71	1460	0.99	0.93–1.05	0.70
2.50 to 4.00	164	0.89	0.76–1.04	0.13	126	0.90	0.75–1.09	0.27
Longitudinal effect								
0 to <1.50	3792	1			3209	1		
1.50 to <2.50	1285	1.05	0.97–1.14	0.20	1059	1.08	0.99–1.17	0.09
2.50 to 4.00	117	1.04	0.84–1.28	0.75	92	1.01	0.79–1.29	0.94
*p* ^d^				0.42				0.59

^a^Mutually adjusted cross-sectional and longitudinal effects, no other adjustments

^b^Crude model with adjustment for age, gender, education, income, smoking, alcohol, leisure time physical activity, body mass index, general health, psychiatric disease, cardiovascular disease, disturbed sleep, sleeping hours, daytime work schedule, and sampling on a workday. Morning cortisol was further adjusted for awakening time and sampling time since awakening, and evening cortisol for evening sampling time

^c^Effect ratios [the ratio by which cortisol concentrations (nmol/l) increase by a 1 unit increase in PSS (continuous models) or by a higher PSS category compared to the lowest category (categorical models)] and their 95% confidence intervals (CI)

^d^ *p* value of no difference between cross-sectional and longitudinal effects (Hausman test)

**Fig. 1:**
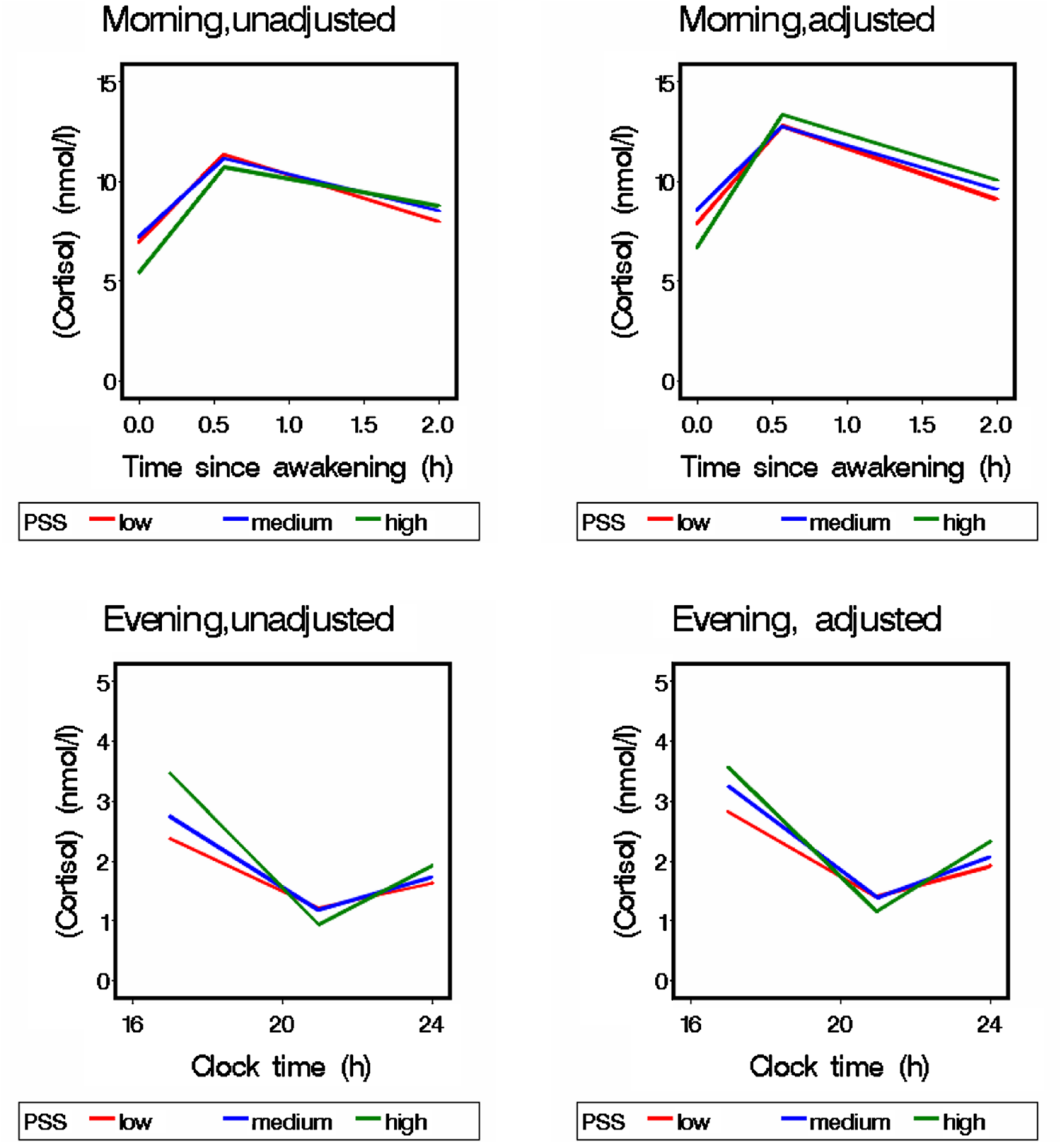
Morning and evening cortisol trajectories by levels of perceived stress scale (PSS) (*low* 0 to <1.50, *medium* 1.50 to <2.50, *high* 2.50 to 4.00). Cross-sectional data 2007 and 2009 combined

The effects of PSS on the relation between sampling times and cross-sectional cortisol are graphically illustrated in Fig. 1. The estimated CAR increased by 70% (95% CL 54–89), 62% (95% CL 36–93), and 108% (95% CL 21–257) by increasing levels of PSS, but the difference was not statistically significant and nor were any other interactions between PSS and the linear slopes of the morning or the evening sampling times (data not shown). When split into cross-sectional and longitudinal effects, the slopes for the different levels of PSS had too wide confidence intervals to be informative. Similarly, there were no statistically significant differences between AUCi’s and AUCt’s for different PSS levels (data not shown).

Higher levels of perceived stress were associated with later sampling times and a larger variation in sampling times than lower levels. For low, medium, and high levels of perceived stress, the respective mean (SD) of saliva sampling times in the morning was 0.63 (0.32), 0.66 (0.34), and 0.69 (0.34) h after awakening in 2007. The corresponding figures for 2009 were 0.63 (0.34), 0.65 (0.35), and 0.66 (0.45) h after awakening, for 2007 evening 20:37 (1.14), 20:38 (1.13), and 20:42 (1.29), and for 2009 evening 20:51 (1.28), 20:50 (1.33), and 21:00 (1.41).

The worst case scenario of the simulation study did not support that differential accuracy in reported sampling times could have biased the estimated main effect of PSS or diminished the power for detecting an effect. However, the interactions between PSS and sampling time slopes could be biased, even substantially, since the power for demonstrating such an interaction was poor.

In contrast, the worst case simulated misclassification due to inaccuracy of the PSS-4 scale attenuated the estimated main effect of PSS by 25%, but due to the large sample size of the study, the power for detecting an effect of PSS remained high.

The results from the sensitivity analyses were very similar to the main analyses (data not shown), indicating that the main results were not overadjusted or influenced by statistical error in the estimation of the optimal inflexion points for the splines.

## Discussion

In this large cohort study of public sector employees, we found no cross-sectional or longitudinal effects of PSS on the level or the time trajectory of the morning or evening cortisol.

### Perceived stress scale

The PSS was first based on 14 items (PSS-14) (Cohen et al. 1983), later reduced to 10 and 4 items (PSS-10 and PSS-4) (Cohen and Willliamson 1988) based on items from the original scale. The psychometric properties of the PSS instrument have recently been reviewed by Lee (2012). She found that the PSS had good internal reliability and test–retest reliability for up to 4 weeks, and that PSS scores were consistently associated with characteristics such as age, income, employment, ethnicity, and marital status.

We used the PSS-4 version of PSS which except from a lower internal reliability seems to have psychometric and predictive properties comparable to PSS-10 (Cohen and Willliamson 1988; Lee 2012). The internal reliability of the PSS-4 scale was the same (0.60) in 2007 and 2009 and comparable to other data (Cohen and Willliamson 1988; Lee 2012). The lower internal reliability of PSS-4 implies an increased statistical uncertainty when estimating the effect of PSS compared to PSS versions with 10 or 14 items (e.g., if PSS-4 has a reliability of 0.60 compared to 0.80 of the PSS-10 scale, then the expected attenuation of the standardized effect when using PSS-4 as a continuous predictor is √(0.60/0.80) = 0.866 or −13.4%) but does not alter the direction of the effect of PSS. This expected power reduction from using PSS-4 is compensated by the size of our study (see the “Results” and Supplementary material, Appendix 1).

The distribution of PSS-4 scores in our study [mean (SD) 2007/2009: 1.1 (0.6)/1.0 (0.6)] was similar to the distribution of PSS-10 scores in a Danish national population sample [mean (SD): 11.0 (5.9), *n* = 10,250] (Stigsdotter et al. 2010) and PSS-4 scores in a USA population sample (Cohen and Willliamson 1988), albeit lower than in a normative English sample (Warttig et al. 2013). For comparisons, note that our PSS score is the mean of item scores, while other studies used the sum of item scores (e.g., PSS-10 sum score is ten times higher than our PPS-4 mean score).

Furthermore, the exposure contrast was large with more persons in PSS categories ‘fairly often’ or ‘very often’ than in any other study on PSS and cortisol (see below), perhaps with one exception (Putterman and Linden 2006). Thus, we have no reason to assume that our null findings may be accounted for by low validity or too little contrast in PSS scores.

### Cortisol

Our cortisol analyses were made with an established and well-documented method (Hansen et al. 2003). However, morning cortisol increased from 2007 to 2009. Selection, changes in cortisol determinants from baseline to follow-up, and a shorter storage time in 2009 explained approximately half of the increase (data not shown). Saliva sampling methods, instructions, sampling times, and laboratory methods were the same across examination rounds. A possible explanation may be a cotton tampon batch change. Cortisol associations to awakening and sampling times and to other determinants of cortisol, including PSS, were very similar in 2007 and 2009. We, therefore, consider the drift in morning cortisol concentrations from 2007 to 2009 as non-differential in relation to perceived stress. In the longitudinal analyses, we adjusted for a temporal drift by controlling for effects of examination round.

### Awakening and saliva sampling times

Owing to economic constraints, we had only one morning and one evening cortisol measurement per participant in each examination round, and had to rely on self-reports on awakening time and saliva sampling times.

The correlation between self-reported and electronically tagged saliva sampling times has been reported to be between 0.75 and 0.90, depending on sampling time points (Karlamangla et al. 2013), and 85% of self-reported awakening times has been reported as correct within 10 min compared to objectively recorded awakening time (Dockray et al. 2008). Inaccurately reported sampling times may bias the estimated relation between cortisol and perceived stress towards the null if the inaccuracy is more pronounced among persons with high levels of perceived stress compared to persons with low levels. However, in our worst case scenario simulation study (Supplementary material, Appendix 1), differential accuracy in reported sampling times did not bias the estimated main effect of PSS and did not diminish the power for detecting the effect. However, we cannot rule out that PSS affects the time course of cortisol rather than the level, since the power for demonstrating such an interaction was poor.

On average, participants adhered well to sample time instructions, but the large variation in sampling times required an adjustment for the effects of different sampling times. The large variation and a large sample size enabled us to model the time trajectory of morning and evening cortisol in the population, using linear splines. The cross-sectional estimates of the time trajectory in this model are expected to give the same results as if we had replicated within-person measurements at specific time points. This is a property of linear growth models: the mean of the individual slopes is the same as the population mean slope. In particular, the slope of the first linear piece of time since awakening is a valid measure of the mean of individual cortisol awakening responses (CAR). The morning and evening time trajectories of cortisol secretion were very similar for 2007 and 2009, and they were in good accordance with the previous large epidemiological studies, e.g., (Karlamangla et al. 2013; Ranjit et al. 2005) and the well-known diurnal pattern of cortisol secretion (Supplementary material, Appendix 1). The validity of our measures of the diurnal variation in cortisol is further supported by significant associations with a number of covariates which have previously been associated with saliva cortisol concentrations, e.g., sampling on a workday versus on a day off, smoking, and physical activity (data not shown).

### Confounding

The size of our study allowed adjustment for effects of a large set of potential confounders. However, the crude associations did not change much by this adjustment, and therefore, it seems less likely that other unknown factors not accounted for would do so. This assumption is further supported by the fact that cross-sectional and longitudinal effects of PSS on cortisol did not differ significantly as they most likely would in the presence of important unmeasured confounders (Fitzmaurice et al. 2011). The similarity between crude and adjusted results also speaks against overadjustment. The issue of overadjustment was also dealt with directly by excluding factors which could be antecedent (e.g., life events) or intermediate factors (e.g., life style and sampling times) in a causal path from perceived stress to cortisol. We did not instruct participants to avoid specific behaviors in relation to saliva sampling, e.g., tooth brushing, eating, drinking, smoking, or physical activity, because we assumed that such restrictions could reduce participation. Furthermore, there is no consistent evidence that saliva cortisol is influenced by tooth brushing, drinking caffeinated drinks, or ordinary physical activity (Kudielka et al. 2009; Stalder et al. 2016). Eating and drinking soft drinks before taking the saliva samples may have caused transient increases in saliva cortisol. It seems unlikely, however, that pre-sampling eating and drinking would be more common among participants with low than with high PSS, which has to be the pattern to bias our results towards null. This condition also applies to other uncontrolled factors if they should act as confounders (e.g., use of oral glucocorticoid medication).

### Selection

Non-response at baseline and loss to follow-up in 2009 could potentially affect our results if non-participation was associated with PSS and this selection was associated with cortisol. Among participants in 2007 who also participated in 2009, the PSS was on average 0.08 scale scores lower than those who did not participate in 2009 (*p* < 0.001), but neither morning nor evening cortisol concentrations were associated with this selection. Furthermore, there were no significant differences in PSS effects on cortisol in the cross-sectional data from 2007 and 2009 which also speaks against selection bias as an explanation for the results.

### Prevalence of potentially harmful stress

According to the theory of allostatic load frequent acute stress may cause prolonged exposure to stress hormones, which in turn is harmful to health (Chrousos and Kino 2007; McEwen 1998). The term ‘frequent’ is ambiguous, but could apply to PSS categories ‘fairly often’ and ‘very often’, but hardly to the PSS category ‘sometimes’. Based on this consideration, the 1 month prevalence of potentially harmful stress was approximately 2.5% in our study. Only 14 participants (0.5%) had PSS scores indicating that perceived stress was experienced ‘fairly often’ or ‘very often’ in both examination rounds. Thus, the prevalence of potentially harmful stress over prolonged periods seems to be somewhere between 0.5 and 2.5% in this Danish public service population when measured by the PSS instrument. This level of PSS was not associated with cortisol in the cross-sectional data, and in the longitudinal data, a change to or from this level was not associated with any change in cortisol.

### Other studies

Based on a previous literature review of 18 studies (Halford et al. 2011), a supplemental literature search, and hand searching references in this literature, we identified 28 studies with data on the relation between PSS-10 or PSS-14 (no PSS-4 studies were found) and some aspect of cortisol secretion [single sample measures at different times of the day, several samples across the day assessed separately or in combination, e.g., as the CAR or area under the curve (AUC)] (Abercrombie et al. 2004; Bohbot et al. 2011; Camfield et al. 2013; Edwards et al. 2003; Farag et al. 2008; Gallagher-Thompson et al. 2006; Groeneveld et al. 2012; Izawa et al. 2012; Klatt et al. 2009; Lasikiewicz et al. 2008; Lovell et al. 2011; Mikolajczak et al. 2010; Mondelli et al. 2010; Murphy et al. 2010; Nicolson and van 2000; O’Connor et al. 2009; Ockenfels et al. 1995; Pruessner et al. 1999; Putterman and Linden 2006; Schulze et al. 2009; Schwarz and Dunphy 2003; Simpson et al. 2008; Stalder et al. 2011; Thorn et al. 2006; Tull et al. 2005; Turner-Cobb et al. 2010; van Eck et al. 1996; Wahbeh et al. 2008).

The median number of participants in these studies was 47, and only four studies had over 100 participants (maximum 170). The mean PSS-10 score or median PSS-10 score (or PSS-14 scores converted to PSS-10 scores) was mostly between 10 and 29 (median 17 with standard deviations of approximately 5). Most studies were based on healthy adults recruited in various ways.

In studies of unselected populations (e.g., Camfield et al. 2013; Lovell et al. 2011), the distribution of PSS was skewed with a high probability of low scores and a low probability of high scores, consistent with our results.

None of the studies specified the number of participants with item responses corresponding to ‘fairly often’ or ‘very often’. However, based on the published means/medians and standard deviations, most of these studies seem to have included only few participants reporting perceived stress ‘fairly often’ or ‘very often’, even in groups defined as ‘high stress’ groups based on percentile cutoffs. Rather, these groups have predominantly consisted of participants with perceived stress occurring only ‘sometimes’ during the last month. There were a few exceptions with remarkably high PSS scores, unexplained in two population-based studies (Putterman and Linden 2006; Tull et al. 2005), and in one study probably explained by participants being recruited to a stress-intervention program (Klatt et al. 2009). Some studies did not report adequate PSS distribution statistics (Groeneveld et al. 2012; Izawa et al. 2012; Mikolajczak et al. 2010; O’Connor et al. 2009; Schulze et al. 2009). Fourteen of the studies found no relations between PSS and cortisol measures, including two of the three studies with remarkably high PSS scores (Klatt et al. 2009; Putterman and Linden 2006), and 14 studies found significant associations with some cortisol secretion measure. However, the results were not consistent across specific cortisol measures (e.g., morning or evening, CAR, or AUC). Thus, the results of previous studies are inconsistent with limited support of a relation between PSS and cortisol secretion. It is possible that the null-associations in these studies may be due to too little exposure contrast and too few participants with frequently perceived stress. However, the contrast was generally not any larger in studies finding positive associations. Moreover, our study, which was based on both a larger number of participants and a larger exposure contrast than any previous studies, also generated null findings.

### Strengths and limitations

It is a limitation of our study that we had only two cortisol measurement points. This limitation, however, was significantly reduced, because participants took their saliva samples across a large time span. This sampling time behavior and the size of the study enabled us to estimate the morning and evening cortisol time trajectories and to assess if PSS changed the cortisol level or the slopes of these trajectories, which is a major strength of the study. It is a limitation that we had to rely on self-reported awakening and saliva sampling times, but this limitation is to some extent inherent in large-scale epidemiological studies for economic and logistic reasons. However, our data did not support that inaccuracies in reported sampling times have biased our results. A further limitation is the use of PSS-4 with a lower internal reliability than PSS-10. This limitation, however, was counteracted by the size of the study. It is also a limitation that the time course of PSS from baseline to follow-up is unknown. The major strengths of the study are the size of the study and two examination rounds, which allowed assessment of the stability of cross-sectional findings; longitudinal analyses enhancing causal inferences; inclusion of a substantial number of persons with frequently perceived stress (PSS ≥ 2.50); and adjustment for a large set of potential confounders.

### Interpretation

Altogether, our results were at odds with expectations based on the assumption that frequently perceived stress would be associated with an increased exposure to cortisol or a change in the diurnal cortisol trajectory. The PSS was designed to measure the degree to which situations in one’s life are appraised as unpredictable, uncontrollable, and overloading (Cohen et al. 1983). This description seems consistent with descriptions of psychosocial exposures perceived as threatening or excessively demanding and, therefore, suited to elicit a response from the HPA system with increased cortisol secretion (Allen et al. 2014).

However, the exposures queried about in the PSS do not have an obvious acute character. The 1 month prevalence period for PSS may be too short to induce longer lasting changes in the HPA-axis regulation of cortisol secretion. We do not know for how long periods frequently perceived stress (PSS ≥ 2.50) was present, but after 2 years approximately 80% of participants with frequently perceived stress at baseline had changed to lower levels of PSS. Neither do we know for how long periods a certain level of frequently repeated acute stress or a sustained level of stress must be to induce longer lasting changes in HPA-axis regulation of cortisol secretion. In comparison, low socioeconomic status entails a multitude of stressful exposures (Gustafsson et al. 2010) lasting many years, but even this exposure is not consistently related to cortisol (Dowd et al. 2009).

It is comforting that perceived stress in an unselected population of working persons is not associated with hypercortisolism or other changes in normal cortisol secretion. However, these results may not be extrapolated to other measures of perceived stress, other populations, or other settings.

Longitudinal studies on acute and long-term effects of frequently repeated exposures to well-defined significant stressors are needed to better understand the concept of chronic or prolonged perceived stress and its physiologic correlates.

## Conclusion

In this large cohort study, PSS had no cross-sectional or longitudinal effects on the level or time trajectory of the morning or evening cortisol. The 1 month prevalence of frequently perceived stress was low, approximately 2.5%.

## Electronic supplementary material

Below is the link to the electronic supplementary material.

Supplementary material 1 (PDF 399 kb)

Supplementary material 2 (PDF 273 kb)
