# Supplementary material for: Prolonged perceived stress and saliva cortisol in a large cohort of Danish public service employees: cross-sectional and longitudinal associations
Source: Int Arch Occup Environ Health. 2017 Jul 11;90(8):835–48. doi: 10.1007/s00420-017-1241-z (PMC5640736; doi:10.1007/s00420-017-1241-z)
Supplement: Supplementary file 1 — Supplementary material 1 (PDF 399 kb) [file 420_2017_1241_MOESM1_ESM.pdf]

Online supporting information for the following article published in *International Archives of Occupational and Environmental Health*

Prolonged perceived stress and saliva cortisol in a large cohort of Danish public service employees: cross-sectional and longitudinal associations

Sigurd Mikkelsen[1], Julie Lyng Forman[2], Samuel Fink[1], Marianne Agergaard Vammen[1], Jane Frølund Thomsen[1], Matias Brødsgaard Grynderup[3], Åse Marie Hansen[3, 4], Linda Kaerlev[5, 6], Henrik Albert Kolstad[7], Reiner Rugulies[3, 4, 8], Jens Peter Bonde[1]

[1]Department of Occupational and Environmental Medicine, Bispebjerg University Hospital, Copenhagen Denmark

[2]Section of Biostatistics, Department of Public Health, University of Copenhagen, Copenhagen, Denmark

[3] Department of Public Health, University of Copenhagen, Denmark

[4] National Research Centre for the Working Environment, Copenhagen, Denmark

[5] Research Unit of Clinical Epidemiology, Institute of Clinical Research, University of Southern Denmark, Odense, Denmark

[6] Center for Clinical Epidemiology, Odense University Hospital, Odense, Denmark

[7] Department of Occupational Medicine, Danish Ramazzini Centre, Aarhus University Hospital, Århus, Denmark

[8] Department of Psychology, University of Copenhagen, Denmark

Appendix 1: Potential attenuation bias

1) Potential attenuation bias due to inaccuracy in reported sampling time.

It is well known that error in the explanatory variable causes attenuation bias in the effect of that variable. Thus a concern in the PRISME-study is that persons with high stress levels gave less accurate reports on cortisol sampling times than those with lower levels of stress as described in the results section. E.g. in 2007, the reported morning sampling time for persons with PSS levels 1.5-2.5 and 2.5-4.0 were on average 1.8 and 3.6 minutes, respectively, later than for participants with a PSS level <1.5, and sampling times varied slightly more among the persons with higher levels, SD=0.32 hours for PSS<1.5 vs. 0.34 hours for both of the two higher levels.

We conducted a simulation study to investigate the magnitude of the potential attenuation bias and to discuss its implications for the estimated effect of PSS on cortisol. For simplicity we simulated a two-group cross-sectional comparison of a high PSS group (n=1500) vs a normal/low group (n=3000), similar to the distribution of PSS scores <1.50 and ≥1.50 in the study. We chose sample sizes that matched the effective sample size of the study (discounting replicates and degrees of freedom lost due to adjusting covariates). Initial sampling times were simulated from a chi-square distribution with mean 0.63 hours and SD=0.32 hours matching the reported sampling times for PSS<1.5. Normally distributed random errors were added to the sampling times of the high PSS group, thereby increasing the mean to 0.66 and the SD to 0.34. This corresponds to a worst case scenario where excess mean and variance is solely due to reporting error (in reality de facto later sampling is a likely part of the explanation). To further mimic a supposedly realistic reporting of sampling times a random selection of 30% and 20% of the sampling times where rounded to the nearest 5 or 10 minutes and the remaining 50% to the minute. These choices were arbitrary. A previous study has shown that 85% of self-reported awakening times has been reported as correct within 10 minutes compared to objectively recorded awakening time {Dockray, 2008 3899 /id}. Cortisol response was simulated as log-normally distributed with a mean value depending on PSS and initial sampling time, i.e. before adding error to the sampling times in the high PSS group and before rounding in both groups. We used the spline parameters for PSS<1.5 (peak at 34 minutes, peak value 2.50, ascending slope 0.90, and descending slope -0.15), and a residual variance of 0.42, similar to the study.

We considered three different scenarios for the effect of PSS: 1) no effect, 2) 10% increase, and 3) 10% decrease in level of cortisol. In each scenario 10.000 dataset were generated and analyzed with two different spline models one only including a main effect of PSS, the other including interaction terms (i.e. where spline parameters were allowed to differ between the two groups). We compared the estimated parameters to the ‘truth’ and computed the bias in the estimates for the high PSS-group with 90% normal

range. The power / level of the test of main effect of PSS were computed and likewise the power for the test of interaction.

Results

Interestingly hardly any bias is found when estimating the main effect of PSS in any of the three scenarios (table S1). In scenario 2, the power for detecting a 10% increase or decrease in cortisol level in the high PSS is 99.7% in spite of the attenuation. Assuming no effect of PSS, the estimated type I error rate is inflated though only to 5.8%. In all three scenarios the bias is more pronounced in the interaction model where the spline in the high PSS group has been flattened compared to its true shape which is parallel to the low PSS group (figure S1). However, the power for detecting the resulting interaction is merely 21.1% in all three scenarios.

Comparing to the study data, we have seen a similarly decreased peak value in the high PSS group, and also a weak attenuation in the two slope estimates, but the test of interaction was insignificant (P=0.62).

Table S1. Expected attenuation biases due to inaccuracy in reported sampling times.

	**Scenario 1**<br>**True effect "none"**	**Scenario 2**<br>**True effect +10%**	**Scenario 3**<br>**True effect -10%**
**Main effect of PSS**			
Bias in main effect estimate[a]	-0.4% (-3.8%;3.0%)	-0.4% (-3.7%;3.1%)	-0.4% (-3.8%;3.0%)
Level/power of test[b]	5.8%	99.7%	99.9%
**PSS-sampling time interaction**			
Bias in peak value[a]	-0.04 (-0.09;0.01)	-0.04 (-0.09;0.01)	-0.04 (-0.08;0.01)
Bias in ascending slope[a]	-0.24 (-0.48;0.01)	-0.24 (-0.48;0.00)	-0.24 (-0.48;0.00)
Bias in descending slope[a]	0.09 (-0.04;0.21)	0.09 (-0.04;0.21)	0.09 (-0.04;0.22)
Interaction/power of test[b]	21.1%	21.1%	21.1%

[a] Median (90% normal range), from 10.000 simulated datasets. The small differences in attenuation biases between the different scenarios are due to simulation inaccuracies. [b] Proportion of nulls rejected.

Fig S1. Attenuation bias due to inaccurary in reported sampling times (scenario 2).

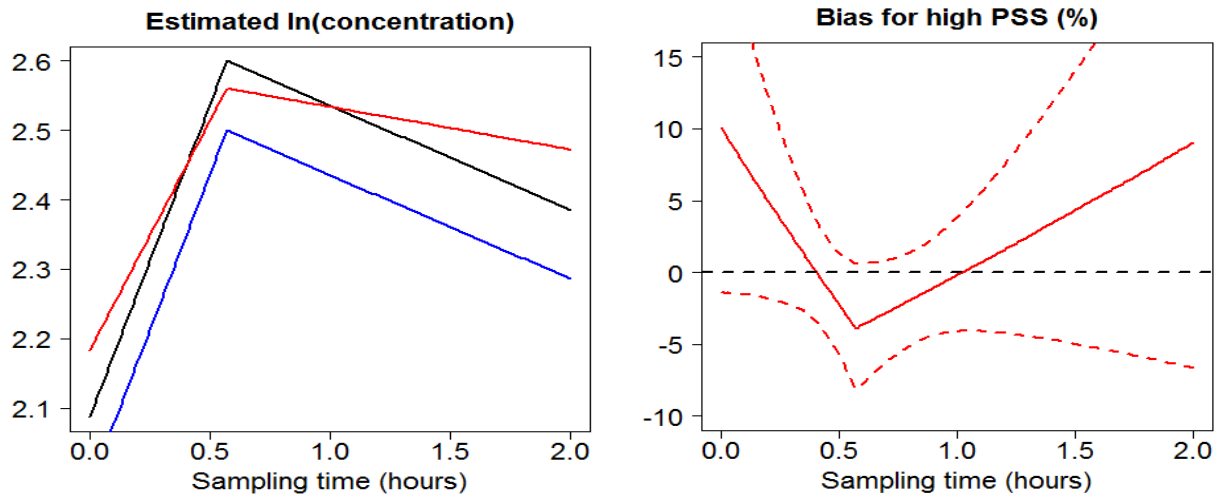

Fig S1: A 10% increased level of cortisol is assumed in the high PSS group (left black curve) compared to the low PSS group (left blue curve). Less accurate reporting of sampling time in the high PSS group result in attenuation of the estimated cortisol response curve (left red curve) compared to the truth and the low PSS group. The expected bias is shown in the right picture (solid line: median, dashed lines: 90% normal range). Based on 10.000 simulated datasets.

2) Potential attenuation bias due to inaccuracy in the PSS4-scale.

To assess the effect of inaccuracy in the PSS4-scale in conjecture with the inaccuracy in reported sampling times we repeated scenario 2 of the simulation study with the addition that 10% (n=450) of the PSS-measurements were misclassified. Specifically we simulated that 250 persons from the low PSS group were misclassified as high, and 200 from the high group were misclassified as low. In the simulation the true effects was set to 10%, but the estimated effect was 7.48%, corresponding to a -2.52% bias or a 25.2% attenuation in the estimated effect of PSS. The power for detecting the difference between the high and low PSS group was 95.0%. In this scenario the slopes of both the high and the low PSS group were

attenuated, implying that the interaction is somewhat less pronounced (figure S2) and the power for detecting it has decreased to 13.9%.

Figure S2. Attenuation due to inaccurary in reported sampling times and misclassified PSS.

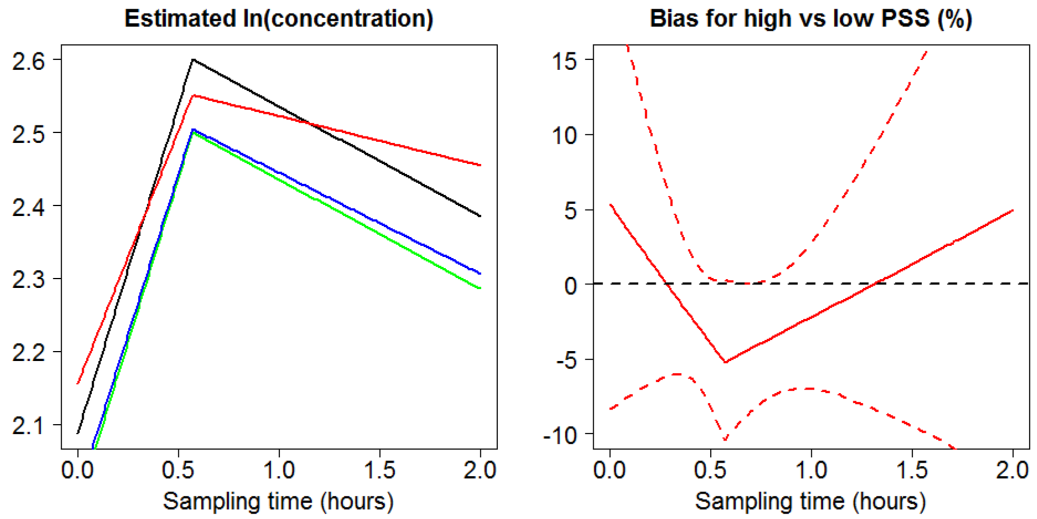

Figure S2: A 10% increased level of cortisol is assumed in the high PSS group (left black curve) compared to the low PSS group (left blue curve). Misclassification into the two groups lead to attenuation of the PSS-effect and less accurate reporting of sampling time in the high PSS group result in attenuation of the estimated cortisol response curves (left red and green curves). The expected bias in estimated difference between the two groups is shown in the right picture (solid line: median, dashed lines: 90% normal range). Based on 10.000 simulated datasets

Conclusions

Based on simulations from a suggested worst case scenario, we conclude that differential accuracy in reported sampling times, does not bias the estimated main effect of PSS and does not diminish the power for detecting the effect. However, we cannot rule out that PSS affect the time course of cortisol rather than the level, since the power for demonstrating such an interaction is poor and a potential differential sampling error could bias relevant estimates substantially. Misclassification due to inaccuracy of the PSS4-

scale, lead to bias in the estimated effect of PSS, but due to the large sample size of the study the power for detecting an effect of PSS is nevertheless high.
