# Supplementary material for: Prolonged perceived stress and saliva cortisol in a large cohort of Danish public service employees: cross-sectional and longitudinal associations
Source: Int Arch Occup Environ Health. 2017 Jul 11;90(8):835–48. doi: 10.1007/s00420-017-1241-z (PMC5640736; doi:10.1007/s00420-017-1241-z)
Supplement: Supplementary file 2 — Supplementary material 2 (PDF 273 kb) [file 420_2017_1241_MOESM2_ESM.pdf]

Appendix 2: Diurnal cortisol trajectory

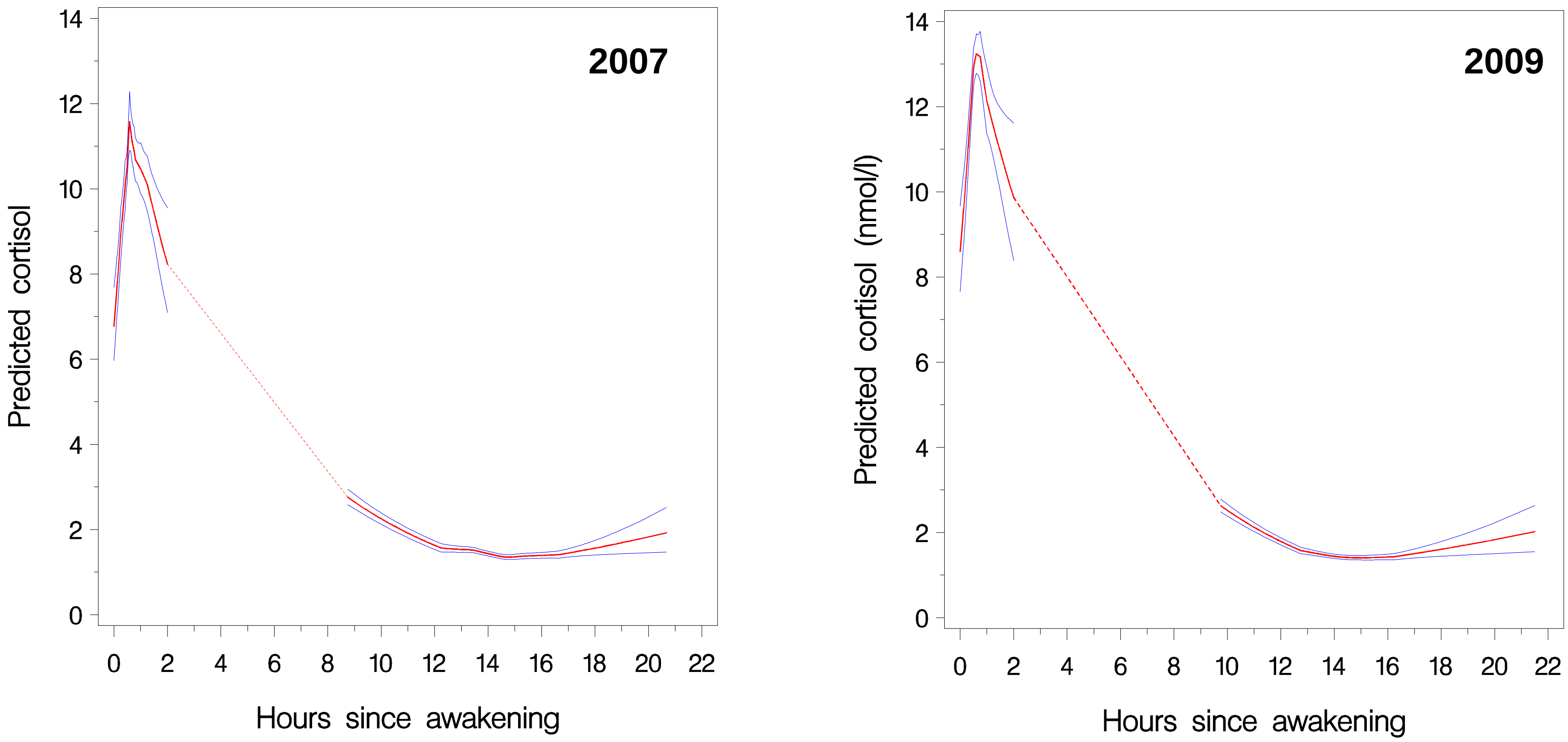

Fig. S2 Cortisol concentrations by hours since awakening (no data between 2 and 10 hours since awakening). Loess-smoothed plots (2007: n=3,633; 2009: n=2,505).
